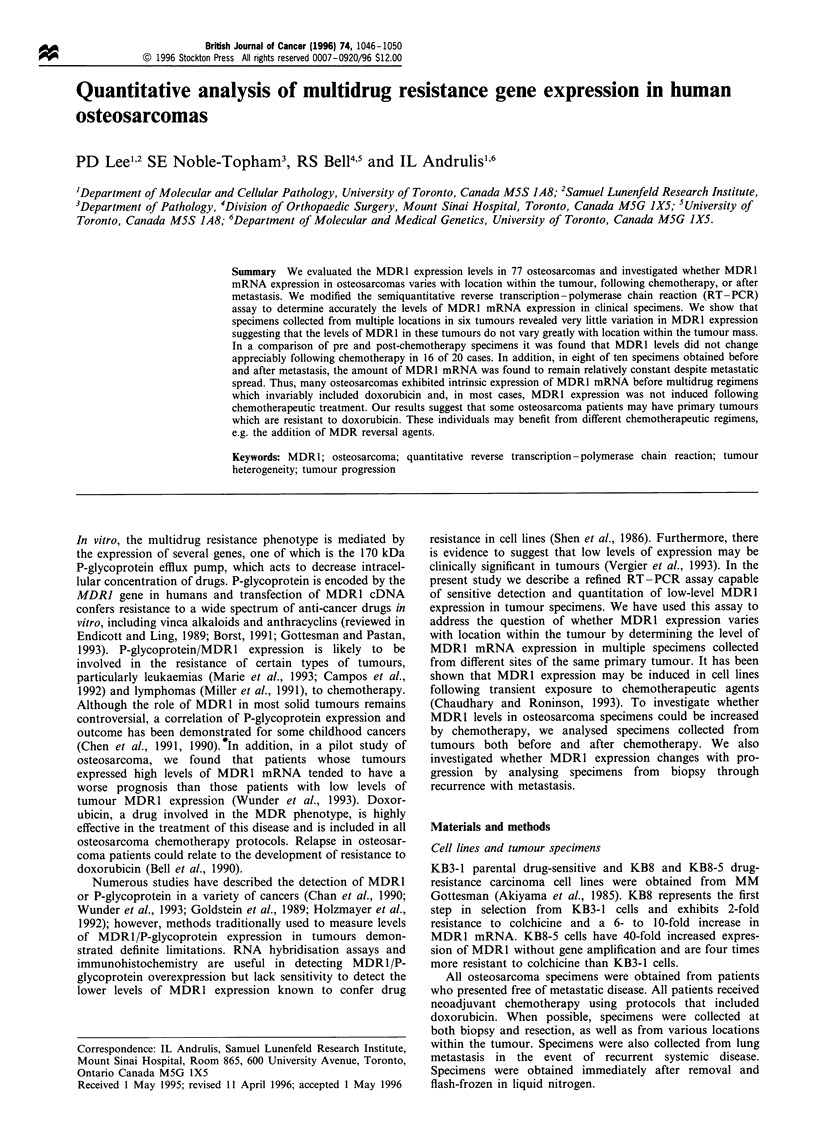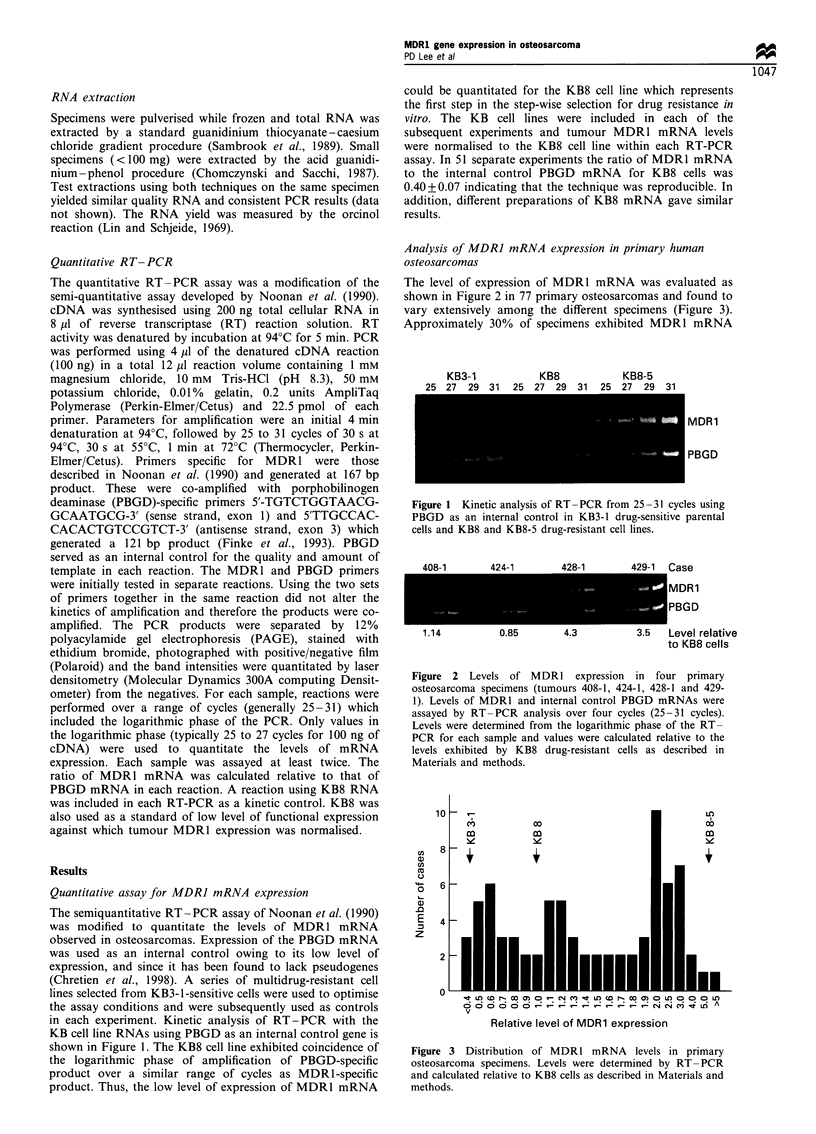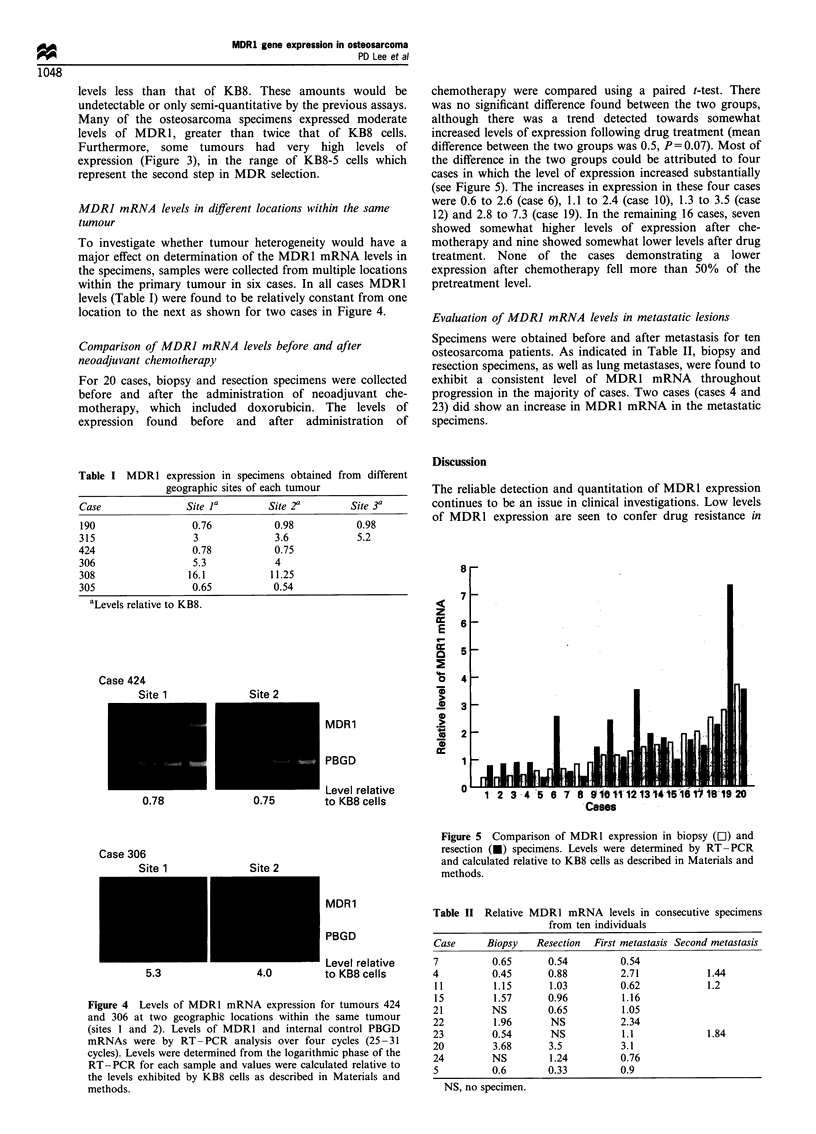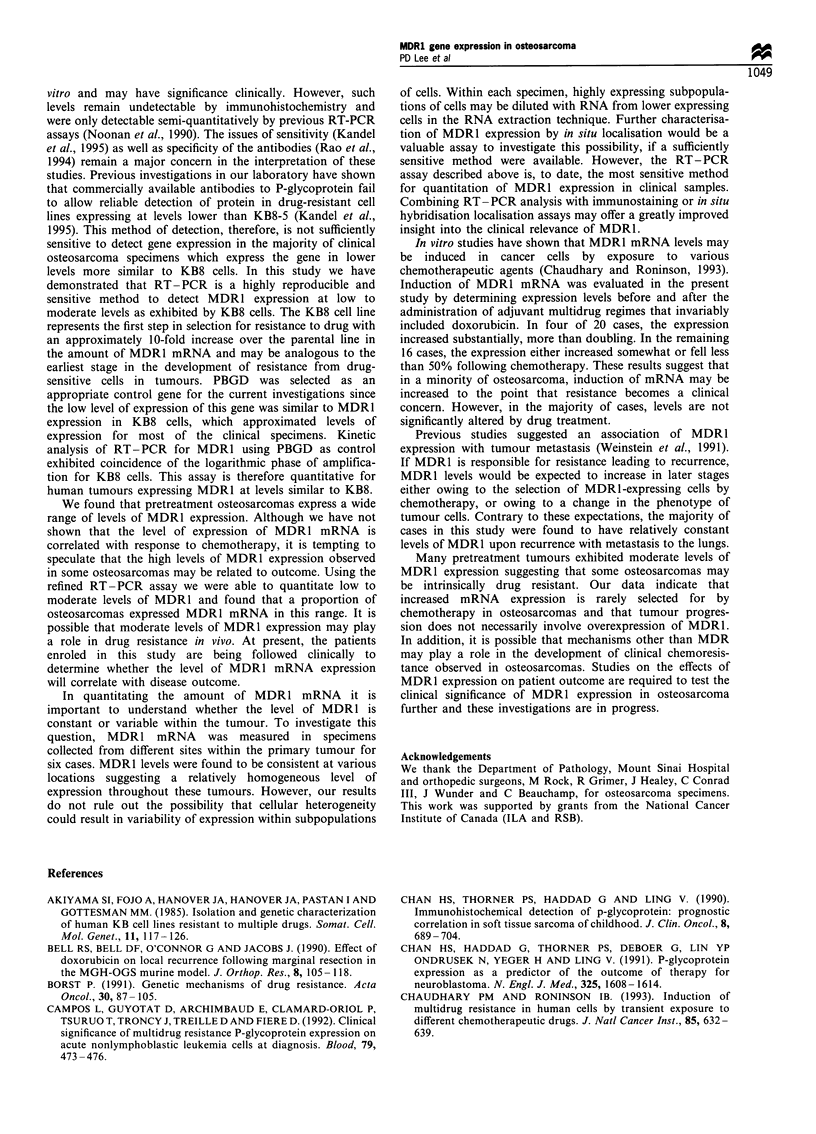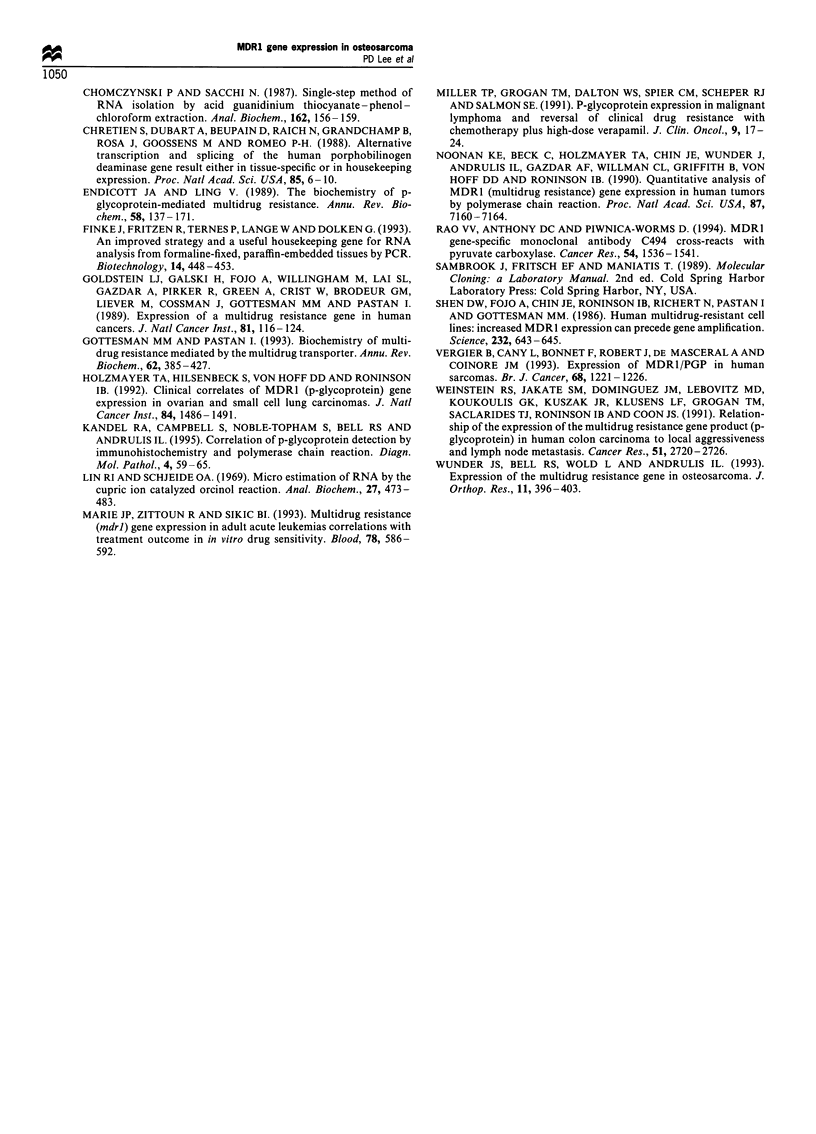# Quantitative analysis of multidrug resistance gene expression in human osteosarcomas.

**DOI:** 10.1038/bjc.1996.487

**Published:** 1996-10

**Authors:** P. D. Lee, S. E. Noble-Topham, R. S. Bell, I. L. Andrulis

**Affiliations:** Department of Molecular and Cellular Pathology, University of Toronto, Canada.

## Abstract

**Images:**


British Journal of Cancer (1996) 74, 1046–1050
© 1996 Stockton Press All rights reserved 0007–0920/96 $12.00

### Quantitative analysis of multidrug resistance gene expression in human osteosarcomas

PD Lee[1,2] SE Noble-Topham[3], RS Bell[4,5] and IL Andrulis[1,6]

[1]*Department of Molecular and Cellular Pathology, University of Toronto, Canada M5S 1A8;* [2]*Samuel Lunenfeld Research Institute,* [3]*Department of Pathology,* [4]*Division of Orthopaedic Surgery, Mount Sinai Hospital, Toronto, Canada M5G 1X5;* [5]*University of Toronto, Canada M5S 1A8;* [6]*Department of Molecular and Medical Genetics, University of Toronto, Canada M5G 1X5.*

**Summary** We evaluated the MDR1 expression levels in 77 osteosarcomas and investigated whether MDR1 mRNA expression in osteosarcomas varies with location within the tumour, following chemotherapy, or after metastasis. We modified the semiquantitative reverse transcription–polymerase chain reaction (RT–PCR) assay to determine accurately the levels of MDR1 mRNA expression in clinical specimens. We show that specimens collected from multiple locations in six tumours revealed very little variation in MDR1 expression suggesting that the levels of MDR1 in these tumours do not vary greatly with location within the tumour mass. In a comparison of pre and post-chemotherapy specimens it was found that MDR1 levels did not change appreciably following chemotherapy in 16 of 20 cases. In addition, in eight of ten specimens obtained before and after metastasis, the amount of MDR1 mRNA was found to remain relatively constant despite metastatic spread. Thus, many osteosarcomas exhibited intrinsic expression of MDR1 mRNA before multidrug regimens which invariably included doxorubicin and, in most cases, MDR1 expression was not induced following chemotherapeutic treatment. Our results suggest that some osteosarcoma patients may have primary tumours which are resistant to doxorubicin. These individuals may benefit from different chemotherapeutic regimens, e.g. the addition of MDR reversal agents.

**Keywords:** MDR1; osteosarcoma; quantitative reverse transcription–polymerase chain reaction; tumour heterogeneity; tumour progression

*In vitro*, the multidrug resistance phenotype is mediated by the expression of several genes, one of which is the 170 kDa P-glycoprotein efflux pump, which acts to decrease intracellular concentration of drugs. P-glycoprotein is encoded by the *MDR1* gene in humans and transfection of MDR1 cDNA confers resistance to a wide spectrum of anti-cancer drugs *in vitro*, including vinca alkaloids and anthracyclins (reviewed in Endicott and Ling, 1989; Borst, 1991; Gottesman and Pastan, 1993). P-glycoprotein/MDR1 expression is likely to be involved in the resistance of certain types of tumours, particularly leukaemias (Marie *et al.*, 1993; Campos *et al.*, 1992) and lymphomas (Miller *et al.*, 1991), to chemotherapy. Although the role of MDR1 in most solid tumours remains controversial, a correlation of P-glycoprotein expression and outcome has been demonstrated for some childhood cancers (Chen *et al.*, 1991, 1990). In addition, in a pilot study of osteosarcoma, we found that patients whose tumours expressed high levels of MDR1 mRNA tended to have a worse prognosis than those patients with low levels of tumour MDR1 expression (Wunder *et al.*, 1993). Doxorubicin, a drug involved in the MDR phenotype, is highly effective in the treatment of this disease and is included in all osteosarcoma chemotherapy protocols. Relapse in osteosarcoma patients could relate to the development of resistance to doxorubicin (Bell *et al.*, 1990).

Numerous studies have described the detection of MDR1 or P-glycoprotein in a variety of cancers (Chan *et al.*, 1990; Wunder *et al.*, 1993; Goldstein *et al.*, 1989; Holzmayer *et al.*, 1992); however, methods traditionally used to measure levels of MDR1/P-glycoprotein expression in tumours demonstrated definite limitations. RNA hybridisation assays and immunohistochemistry are useful in detecting MDR1/P-glycoprotein overexpression but lack sensitivity to detect the lower levels of MDR1 expression known to confer drug resistance in cell lines (Shen *et al.*, 1986). Furthermore, there is evidence to suggest that low levels of expression may be clinically significant in tumours (Vergier *et al.*, 1993). In the present study we describe a refined RT–PCR assay capable of sensitive detection and quantitation of low-level MDR1 expression in tumour specimens. We have used this assay to address the question of whether MDR1 expression varies with location within the tumour by determining the level of MDR1 mRNA expression in multiple specimens collected from different sites of the same primary tumour. It has been shown that MDR1 expression may be induced in cell lines following transient exposure to chemotherapeutic agents (Chaudhary and Roninson, 1993). To investigate whether MDR1 levels in osteosarcoma specimens could be increased by chemotherapy, we analysed specimens collected from tumours both before and after chemotherapy. We also investigated whether MDR1 expression changes with progression by analysing specimens from biopsy through recurrence with metastasis.

Correspondence: IL Andrulis, Samuel Lunenfeld Research Institute, Mount Sinai Hospital, Room 865, 600 University Avenue, Toronto, Ontario Canada M5G 1X5
Received 1 May 1995; revised 11 April 1996; accepted 1 May 1996

### Materials and methods

### *Cell lines and tumour specimens*

KB3-1 parental drug-sensitive and KB8 and KB8-5 drug-resistance carcinoma cell lines were obtained from MM Gottesman (Akiyama *et al.*, 1985). KB8 represents the first step in selection from KB3-1 cells and exhibits 2-fold resistance to colchicine and a 6- to 10-fold increase in MDR1 mRNA. KB8-5 cells have 40-fold increased expression of MDR1 without gene amplification and are four times more resistant to colchicine than KB3-1 cells.

All osteosarcoma specimens were obtained from patients who presented free of metastatic disease. All patients received neoadjuvant chemotherapy using protocols that included doxorubicin. When possible, specimens were collected at both biopsy and resection, as well as from various locations within the tumour. Specimens were also collected from lung metastasis in the event of recurrent systemic disease. Specimens were obtained immediately after removal and flash-frozen in liquid nitrogen.

*RNA extraction*

Specimens were pulverised while frozen and total RNA was extracted by a standard guanidinium thiocyanate–caesium chloride gradient procedure (Sambrook *et al*., 1989). Small specimens (<100 mg) were extracted by the acid guanidinium–phenol procedure (Chomczynski and Sacchi, 1987). Test extractions using both techniques on the same specimen yielded similar quality RNA and consistent PCR results (data not shown). The RNA yield was measured by the orcinol reaction (Lin and Schjeide, 1969).

*Quantitative RT–PCR*

The quantitative RT–PCR assay was a modification of the semi-quantitative assay developed by Noonan *et al.* (1990). cDNA was synthesised using 200 ng total cellular RNA in 8 $\mu$l of reverse transcriptase (RT) reaction solution. RT activity was denatured by incubation at 94°C for 5 min. PCR was performed using 4 $\mu$l of the denatured cDNA reaction (100 ng) in a total 12 $\mu$l reaction volume containing 1 mM magnesium chloride, 10 mM Tris-HCl (pH 8.3), 50 mM potassium chloride, 0.01% gelatin, 0.2 units AmpliTaq Polymerase (Perkin-Elmer/Cetus) and 22.5 pmol of each primer. Parameters for amplification were an initial 4 min denaturation at 94°C, followed by 25 to 31 cycles of 30 s at 94°C, 30 s at 55°C, 1 min at 72°C (Thermocycler, Perkin-Elmer/Cetus). Primers specific for MDR1 were those described in Noonan *et al.* (1990) and generated at 167 bp product. These were co-amplified with porphobilinogen deaminase (PBGD)-specific primers 5′-TGTCTGGTAACG-GCAATGCG-3′ (sense strand, exon 1) and 5′TTGCCAC-CACACTGTCCGTCT-3′ (antisense strand, exon 3) which generated a 121 bp product (Finke *et al*., 1993). PBGD served as an internal control for the quality and amount of template in each reaction. The MDR1 and PBGD primers were initially tested in separate reactions. Using the two sets of primers together in the same reaction did not alter the kinetics of amplification and therefore the products were co-amplified. The PCR products were separated by 12% polyacylamide gel electrophoresis (PAGE), stained with ethidium bromide, photographed with positive/negative film (Polaroid) and the band intensities were quantitated by laser densitometry (Molecular Dynamics 300A computing Densitometer) from the negatives. For each sample, reactions were performed over a range of cycles (generally 25–31) which included the logarithmic phase of the PCR. Only values in the logarithmic phase (typically 25 to 27 cycles for 100 ng of cDNA) were used to quantitate the levels of mRNA expression. Each sample was assayed at least twice. The ratio of MDR1 mRNA was calculated relative to that of PBGD mRNA in each reaction. A reaction using KB8 RNA was included in each RT-PCR as a kinetic control. KB8 was also used as a standard of low level of functional expression against which tumour MDR1 expression was normalised.

### Results

*Quantitative assay for MDR1 mRNA expression*

The semiquantitative RT–PCR assay of Noonan *et al.* (1990) was modified to quantitate the levels of MDR1 mRNA observed in osteosarcomas. Expression of the PBGD mRNA was used as an internal control owing to its low level of expression, and since it has been found to lack pseudogenes (Chretien *et al*., 1998). A series of multidrug-resistant cell lines selected from KB3-1-sensitive cells were used to optimise the assay conditions and were subsequently used as controls in each experiment. Kinetic analysis of RT–PCR with the KB cell line RNAs using PBGD as an internal control gene is shown in Figure 1. The KB8 cell line exhibited coincidence of the logarithmic phase of amplification of PBGD-specific product over a similar range of cycles as MDR1-specific product. Thus, the low level of expression of MDR1 mRNA could be quantitated for the KB8 cell line which represents the first step in the step-wise selection for drug resistance *in vitro*. The KB cell lines were included in each of the subsequent experiments and tumour MDR1 mRNA levels were normalised to the KB8 cell line within each RT-PCR assay. In 51 separate experiments the ratio of MDR1 mRNA to the internal control PBGD mRNA for KB8 cells was 0.40 ± 0.07 indicating that the technique was reproducible. In addition, different preparations of KB8 mRNA gave similar results.

*Analysis of MDR1 mRNA expression in primary human osteosarcomas*

The level of expression of MDR1 mRNA was evaluated as shown in Figure 2 in 77 primary osteosarcomas and found to vary extensively among the different specimens (Figure 3). Approximately 30% of specimens exhibited MDR1 mRNA

**Figure 1** Kinetic analysis of RT–PCR from 25–31 cycles using PBGD as an internal control in KB3-1 drug-sensitive parental cells and KB8 and KB8-5 drug-resistant cell lines.

**Figure 2** Levels of MDR1 expression in four primary osteosarcoma specimens (tumours 408-1, 424-1, 428-1 and 429-1). Levels of MDR1 and internal control PBGD mRNAs were assayed by RT–PCR analysis over four cycles (25–31 cycles). Levels were determined from the logarithmic phase of the RT–PCR for each sample and values were calculated relative to the levels exhibited by KB8 drug-resistant cells as described in Materials and methods.

**Figure 3** Distribution of MDR1 mRNA levels in primary osteosarcoma specimens. Levels were determined by RT–PCR and calculated relative to KB8 cells as described in Materials and methods.

levels less than that of KB8. These amounts would be undetectable or only semi-quantitative by the previous assays. Many of the osteosarcoma specimens expressed moderate levels of MDR1, greater than twice that of KB8 cells. Furthermore, some tumours had very high levels of expression (Figure 3), in the range of KB8-5 cells which represent the second step in MDR selection.

### *MDR1 mRNA levels in different locations within the same tumour*

To investigate whether tumour heterogeneity would have a major effect on determination of the MDR1 mRNA levels in the specimens, samples were collected from multiple locations within the primary tumour in six cases. In all cases MDR1 levels (Table I) were found to be relatively constant from one location to the next as shown for two cases in Figure 4.

### *Comparison of MDR1 mRNA levels before and after neoadjuvant chemotherapy*

For 20 cases, biopsy and resection specimens were collected before and after the administration of neoadjuvant chemotherapy, which included doxorubicin. The levels of expression found before and after administration of chemotherapy were compared using a paired *t*-test. There was no significant difference found between the two groups, although there was a trend detected towards somewhat increased levels of expression following drug treatment (mean difference between the two groups was 0.5, $P=0.07$). Most of the difference in the two groups could be attributed to four cases in which the level of expression increased substantially (see Figure 5). The increases in expression in these four cases were 0.6 to 2.6 (case 6), 1.1 to 2.4 (case 10), 1.3 to 3.5 (case 12) and 2.8 to 7.3 (case 19). In the remaining 16 cases, seven showed somewhat higher levels of expression after chemotherapy and nine showed somewhat lower levels after drug treatment. None of the cases demonstrating a lower expression after chemotherapy fell more than 50% of the pretreatment level.

### *Evaluation of MDR1 mRNA levels in metastatic lesions*

Specimens were obtained before and after metastasis for ten osteosarcoma patients. As indicated in Table II, biopsy and resection specimens, as well as lung metastases, were found to exhibit a consistent level of MDR1 mRNA throughout progression in the majority of cases. Two cases (cases 4 and 23) did show an increase in MDR1 mRNA in the metastatic specimens.

**Table I** MDR1 expression in specimens obtained from different geographic sites of each tumour

| *Case* | *Site 1*[a] | *Site 2*[a] | *Site 3*[a] |
|---|---|---|---|
| 190 | 0.76 | 0.98 | 0.98 |
| 315 | 3 | 3.6 | 5.2 |
| 424 | 0.78 | 0.75 | |
| 306 | 5.3 | 4 | |
| 308 | 16.1 | 11.25 | |
| 305 | 0.65 | 0.54 | |

[a]Levels relative to KB8.

**Figure 4** Levels of MDR1 mRNA expression for tumours 424 and 306 at two geographic locations within the same tumour (sites 1 and 2). Levels of MDR1 and internal control PBGD mRNAs were by RT–PCR analysis over four cycles (25–31 cycles). Levels were determined from the logarithmic phase of the RT–PCR for each sample and values were calculated relative to the levels exhibited by KB8 cells as described in Materials and methods.

### Discussion

The reliable detection and quantitation of MDR1 expression continues to be an issue in clinical investigations. Low levels of MDR1 expression are seen to confer drug resistance *in*

**Figure 5** Comparison of MDR1 expression in biopsy (□) and resection (■) specimens. Levels were determined by RT–PCR and calculated relative to KB8 cells as described in Materials and methods.

**Table II** Relative MDR1 mRNA levels in consecutive specimens from ten individuals

| *Case* | *Biopsy* | *Resection* | *First metastasis* | *Second metastasis* |
|---|---|---|---|---|
| 7 | 0.65 | 0.54 | 0.54 | |
| 4 | 0.45 | 0.88 | 2.71 | 1.44 |
| 11 | 1.15 | 1.03 | 0.62 | 1.2 |
| 15 | 1.57 | 0.96 | 1.16 | |
| 21 | NS | 0.65 | 1.05 | |
| 22 | 1.96 | NS | 2.34 | |
| 23 | 0.54 | NS | 1.1 | 1.84 |
| 20 | 3.68 | 3.5 | 3.1 | |
| 24 | NS | 1.24 | 0.76 | |
| 5 | 0.6 | 0.33 | 0.9 | |

NS, no specimen.

vitro and may have significance clinically. However, such levels remain undetectable by immunohistochemistry and were only detectable semi-quantitatively by previous RT-PCR assays (Noonan *et al.*, 1990). The issues of sensitivity (Kandel *et al.*, 1995) as well as specificity of the antibodies (Rao *et al.*, 1994) remain a major concern in the interpretation of these studies. Previous investigations in our laboratory have shown that commercially available antibodies to P-glycoprotein fail to allow reliable detection of protein in drug-resistant cell lines expressing at levels lower than KB8-5 (Kandel *et al.*, 1995). This method of detection, therefore, is not sufficiently sensitive to detect gene expression in the majority of clinical osteosarcoma specimens which express the gene in lower levels more similar to KB8 cells. In this study we have demonstrated that RT-PCR is a highly reproducible and sensitive method to detect MDR1 expression at low to moderate levels as exhibited by KB8 cells. The KB8 cell line represents the first step in selection for resistance to drug with an approximately 10-fold increase over the parental line in the amount of MDR1 mRNA and may be analogous to the earliest stage in the development of resistance from drug-sensitive cells in tumours. PBGD was selected as an appropriate control gene for the current investigations since the low level of expression of this gene was similar to MDR1 expression in KB8 cells, which approximated levels of expression for most of the clinical specimens. Kinetic analysis of RT-PCR for MDR1 using PBGD as control exhibited coincidence of the logarithmic phase of amplification for KB8 cells. This assay is therefore quantitative for human tumours expressing MDR1 at levels similar to KB8.

We found that pretreatment osteosarcomas express a wide range of levels of MDR1 expression. Although we have not shown that the level of expression of MDR1 mRNA is correlated with response to chemotherapy, it is tempting to speculate that the high levels of MDR1 expression observed in some osteosarcomas may be related to outcome. Using the refined RT-PCR assay we were able to quantitate low to moderate levels of MDR1 and found that a proportion of osteosarcomas expressed MDR1 mRNA in this range. It is possible that moderate levels of MDR1 expression may play a role in drug resistance *in vivo*. At present, the patients enroled in this study are being followed clinically to determine whether the level of MDR1 mRNA expression will correlate with disease outcome.

In quantitating the amount of MDR1 mRNA it is important to understand whether the level of MDR1 is constant or variable within the tumour. To investigate this question, MDR1 mRNA was measured in specimens collected from different sites within the primary tumour for six cases. MDR1 levels were found to be consistent at various locations suggesting a relatively homogeneous level of expression throughout these tumours. However, our results do not rule out the possibility that cellular heterogeneity could result in variability of expression within subpopulations of cells. Within each specimen, highly expressing subpopulations of cells may be diluted with RNA from lower expressing cells in the RNA extraction technique. Further characterisation of MDR1 expression by *in situ* localisation would be a valuable assay to investigate this possibility, if a sufficiently sensitive method were available. However, the RT-PCR assay described above is, to date, the most sensitive method for quantitation of MDR1 expression in clinical samples. Combining RT-PCR analysis with immunostaining or *in situ* hybridisation localisation assays may offer a greatly improved insight into the clinical relevance of MDR1.

*In vitro* studies have shown that MDR1 mRNA levels may be induced in cancer cells by exposure to various chemotherapeutic agents (Chaudhary and Roninson, 1993). Induction of MDR1 mRNA was evaluated in the present study by determining expression levels before and after the administration of adjuvant multidrug regimes that invariably included doxorubicin. In four of 20 cases, the expression increased substantially, more than doubling. In the remaining 16 cases, the expression either increased somewhat or fell less than 50% following chemotherapy. These results suggest that in a minority of osteosarcoma, induction of mRNA may be increased to the point that resistance becomes a clinical concern. However, in the majority of cases, levels are not significantly altered by drug treatment.

Previous studies suggested an association of MDR1 expression with tumour metastasis (Weinstein *et al.*, 1991). If MDR1 is responsible for resistance leading to recurrence, MDR1 levels would be expected to increase in later stages either owing to the selection of MDR1-expressing cells by chemotherapy, or owing to a change in the phenotype of tumour cells. Contrary to these expectations, the majority of cases in this study were found to have relatively constant levels of MDR1 upon recurrence with metastasis to the lungs.

Many pretreatment tumours exhibited moderate levels of MDR1 expression suggesting that some osteosarcomas may be intrinsically drug resistant. Our data indicate that increased mRNA expression is rarely selected for by chemotherapy in osteosarcomas and that tumour progression does not necessarily involve overexpression of MDR1. In addition, it is possible that mechanisms other than MDR may play a role in the development of clinical chemoresistance observed in osteosarcomas. Studies on the effects of MDR1 expression on patient outcome are required to test the clinical significance of MDR1 expression in osteosarcoma further and these investigations are in progress.

**Acknowledgements**
We thank the Department of Pathology, Mount Sinai Hospital and orthopedic surgeons, M Rock, R Grimer, J Healey, C Conrad III, J Wunder and C Beauchamp, for osteosarcoma specimens. This work was supported by grants from the National Cancer Institute of Canada (ILA and RSB).

### References

AKIYAMA SI, FOJO A, HANOVER JA, HANOVER JA, PASTAN I AND GOTTESMAN MM. (1985). Isolation and genetic characterization of human KB cell lines resistant to multiple drugs. *Somat. Cell. Mol. Genet.*, **11**, 117-126.

BELL RS, BELL DF, O'CONNOR G AND JACOBS J. (1990). Effect of doxorubicin on local recurrence following marginal resection in the MGH-OGS murine model. *J. Orthop. Res.*, **8**, 105-118.

BORST P. (1991). Genetic mechanisms of drug resistance. *Acta Oncol.*, **30**, 87-105.

CAMPOS L, GUYOTAT D, ARCHIMBAUD E, CLAMARD-ORIOL P, TSURUO T, TRONCY J, TREILLE D AND FIERE D. (1992). Clinical significance of multidrug resistance P-glycoprotein expression on acute nonlymphoblastic leukemia cells at diagnosis. *Blood*, **79**, 473-476.

CHAN HS, THORNER PS, HADDAD G AND LING V. (1990). Immunohistochemical detection of p-glycoprotein: prognostic correlation in soft tissue sarcoma of childhood. *J. Clin. Oncol.*, **8**, 689-704.

CHAN HS, HADDAD G, THORNER PS, DEBOER G, LIN YP ONDRUSEK N, YEGER H AND LING V. (1991). P-glycoprotein expression as a predictor of the outcome of therapy for neuroblastoma. *N. Engl. J. Med.*, **325**, 1608-1614.

CHAUDHARY PM AND RONINSON IB. (1993). Induction of multidrug resistance in human cells by transient exposure to different chemotherapeutic drugs. *J. Natl Cancer Inst.*, **85**, 632-639.

CHOMCZYNSKI P AND SACCHI N. (1987). Single-step method of RNA isolation by acid guanidinium thiocyanate–phenol–chloroform extraction. *Anal. Biochem.*, **162,** 156–159.

CHRETIEN S, DUBART A, BEUPAIN D, RAICH N, GRANDCHAMP B, ROSA J, GOOSSENS M AND ROMEO P-H. (1988). Alternative transcription and splicing of the human porphobilinogen deaminase gene result either in tissue-specific or in housekeeping expression. *Proc. Natl Acad. Sci. USA*, **85,** 6–10.

ENDICOTT JA AND LING V. (1989). The biochemistry of p-glycoprotein-mediated multidrug resistance. *Annu. Rev. Biochem.*, **58,** 137–171.

FINKE J, FRITZEN R, TERNES P, LANGE W AND DOLKEN G. (1993). An improved strategy and a useful housekeeping gene for RNA analysis from formaline-fixed, paraffin-embedded tissues by PCR. *Biotechnology*, **14,** 448–453.

GOLDSTEIN LJ, GALSKI H, FOJO A, WILLINGHAM M, LAI SL, GAZDAR A, PIRKER R, GREEN A, CRIST W, BRODEUR GM, LIEVER M, COSSMAN J, GOTTESMAN MM AND PASTAN I. (1989). Expression of a multidrug resistance gene in human cancers. *J. Natl Cancer Inst.*, **81,** 116–124.

GOTTESMAN MM AND PASTAN I. (1993). Biochemistry of multidrug resistance mediated by the multidrug transporter. *Annu. Rev. Biochem.*, **62,** 385–427.

HOLZMAYER TA, HILSENBECK S, VON HOFF DD AND RONINSON IB. (1992). Clinical correlates of MDR1 (p-glycoprotein) gene expression in ovarian and small cell lung carcinomas. *J. Natl Cancer Inst.*, **84,** 1486–1491.

KANDEL RA, CAMPBELL S, NOBLE-TOPHAM S, BELL RS AND ANDRULIS IL. (1995). Correlation of p-glycoprotein detection by immunohistochemistry and polymerase chain reaction. *Diagn. Mol. Pathol.*, **4,** 59–65.

LIN RI AND SCHJEIDE OA. (1969). Micro estimation of RNA by the cupric ion catalyzed orcinol reaction. *Anal. Biochem.*, **27,** 473–483.

MARIE JP, ZITTOUN R AND SIKIC BI. (1993). Multidrug resistance (*mdr1*) gene expression in adult acute leukemias correlations with treatment outcome in *in vitro* drug sensitivity. *Blood*, **78,** 586–592.

MILLER TP, GROGAN TM, DALTON WS, SPIER CM, SCHEPER RJ AND SALMON SE. (1991). P-glycoprotein expression in malignant lymphoma and reversal of clinical drug resistance with chemotherapy plus high-dose verapamil. *J. Clin. Oncol.*, **9,** 17–24.

NOONAN KE, BECK C, HOLZMAYER TA, CHIN JE, WUNDER J, ANDRULIS IL, GAZDAR AF, WILLMAN CL, GRIFFITH B, VON HOFF DD AND RONINSON IB. (1990). Quantitative analysis of MDR1 (multidrug resistance) gene expression in human tumors by polymerase chain reaction. *Proc. Natl Acad. Sci. USA*, **87,** 7160–7164.

RAO VV, ANTHONY DC AND PIWNICA-WORMS D. (1994). MDR1 gene-specific monoclonal antibody C494 cross-reacts with pyruvate carboxylase. *Cancer Res.*, **54,** 1536–1541.

SAMBROOK J, FRITSCH EF AND MANIATIS T. (1989). *Molecular Cloning: a Laboratory Manual.* 2nd ed. Cold Spring Harbor Laboratory Press: Cold Spring Harbor, NY, USA.

SHEN DW, FOJO A, CHIN JE, RONINSON IB, RICHERT N, PASTAN I AND GOTTESMAN MM. (1986). Human multidrug-resistant cell lines: increased MDR1 expression can precede gene amplification. *Science*, **232,** 643–645.

VERGIER B, CANY L, BONNET F, ROBERT J, DE MASCERAL A AND COINDRE JM (1993). Expression of MDR1/PGP in human sarcomas. *Br. J. Cancer*, **68,** 1221–1226.

WEINSTEIN RS, JAKATE SM, DOMINGUEZ JM, LEBOVITZ MD, KOUKOULIS GK, KUSZAK JR, KLUSENS LF, GROGAN TM, SACLARIDES TJ, RONINSON IB AND COON JS. (1991). Relationship of the expression of the multidrug resistance gene product (p-glycoprotein) in human colon carcinoma to local aggressiveness and lymph node metastasis. *Cancer Res.*, **51,** 2720–2726.

WUNDER JS, BELL RS, WOLD L AND ANDRULIS IL. (1993). Expression of the multidrug resistance gene in osteosarcoma. *J. Orthop. Res.*, **11,** 396–403.